# Approximating the cost-benefit analysis to a badge system to track hand hygiene in the hospital setting

**DOI:** 10.1017/ash.2026.10304

**Published:** 2026-02-09

**Authors:** David Chan, Suzanne Robertson, Andreas Poon, Gonzalo Bearman

**Affiliations:** Virginia Commonwealth University, USA

## Abstract

**Objective:**

To model the impact of a badge system on hand hygiene and the impact of increased compliance rates on HAIs with the associated cost savings.

**Setting:**

We consider a variable number of targeted beds within a hospital.

**Methods:**

Using a Markov chain model, we estimate the effect of hand hygiene compliance on HAI transmission over the course of a year. Based on a given level of compliance, the estimated savings are also calculated.

**Results:**

With each 10% increment increase in compliance, we estimate a decrease in approximately 7.5 HAIs per year per 100 targeted beds. A 10% increase above baseline in compliance also results in around $100,000 cost savings per year per 100 targeted beds.

**Conclusions:**

Due to the relative low cost of implementation and upkeep to the badge system, the reduction in HAIs and increase in cost savings make the badge system a worthwhile addition.

## Introduction

Hospital-acquired infections (HAI) are responsible for an estimated annual cost ranging between $28.4 billion to $45.0 billion in the United States.^1^ Many HAIs are caused by multidrug resistant organisms, which may lead to infections such as: central line-associated bloodstream infections (CLABSI), surgical site infections (SSI), catheter-associated urinary tract infections (CAUTI), and ventilator-associated pneumonia (VAP).^2^ Approximately one in every 31 patients have one or more HAIs, leading to an estimated total of 687,000 HAIs, and 78,000 patient deaths in 2015.^3^ One strategies for reducing the spread of HAIs is through increasing hand hygiene(HH) compliance and awareness of the 5 moments of (HH).^4^ Promoting (HH) can be done through interventional methods such as direct and indirect observation, promotional ads, alcohol hand rub dispensers, electronic badges, electronic monitoring, and notification systems.^4–6^

Here we will focus on hospital acquired bloodstream infections, which we will simply refer to as HA-BSIs, which we define as a new blood infection occurring in a patient being in a hospital after 48 hours. We attempt to quantify the decrease in HA-BSIs due to increased (HH) compliance through implementing an electronic badge system.

The quantity and rates of acquiring HA-BSIs can be reduced through intervention strategies that increase (HH) compliance rates. Electronic hand hygiene monitoring systems (EHHMS), specifically with badge sensors, have shown positive results for increasing compliance of hand washing, and reducing HAI rates. Earliest recorded uses of the badge system occurred in 2012, with badges developed by Biovigil.^7^ The EHHMS badge system involves placing a Bluetooth tag on the name badge of each healthcare worker to identify when a (HH) action has taken place in relation to a possible HH opportunity.^8^ These actions are registered by placing sensors on soap and alcohol hand rub dispensers that communicate with the badge.^8^ One study from 2016–2020, using electronic badges, saw an increase in (HH) compliance from 89.8% to 97.1%, and a decrease in *C. difficile* rates from 9.5 to 3.7 per 10,000 patient days.^9^

The following study used a Markov chain model to estimate the impact of implementing a badge system within the hospital setting to improve handwashing compliance. This model assumed an additive increase in HA-BSI

## Methods

A Markov chain model was constructed to estimate the number of HA-BSIs occurring within the hospital setting based on the probability of individuals complying with hand washing during a typical day. This model assumed that each patient is visited an average number of *k* times on a typical day with an opportunity for healthcare workers to wash their hands before each visit. The likelihood of transmitting an HAI is based on the number of consecutively missed handwashing opportunities prior to each visit. It is also assumed that each missed handwashing opportunity increases the likelihood of conveying an HAI from the healthcare staff to a patient. This increase is assumed to be additive.

To construct the Markov chain model, let *P*(*n*,*i*) and *H*(*n,i*) denote the number of patients without and with an HA-BSI on day n after *i* visits, respectively. Let ρ be the base probability of a healthcare worker with washed hands conveying an HA-BSI to a patient. We denote γ as the amount of increase in the probability of conveying an HA-BSI to a patient for each handwashing opportunity missed, for example if a healthcare worker misses 3 handwashing opportunities, then the probability of conveying an HA-BSI would be *ρ* + 3*γ*.

The average compliance rate of healthcare workers within the hospital is denoted by *c,* where *c* is between 0 and 1. This rate is used to estimate the number of handwashing opportunities missed directly preceding the *i*th visit by a healthcare worker to a patient. Thus for the *i*th visit by a healthcare worker, a fraction *c* have just washed their hands, (1 – *c*)*c* have missed washing their hands once, (1 – *c*)^2^*c* have missed washing their hands twice, …, and (1 – *c*)^*i*^ have missed washing their hands all *i*-times. This leads to the probability of conveying an HAI on the *i*th visit to be *cρ* for healthcare workers who just washed their hands, (1 – *c*)c(*ρ* + *γ*) for those who missed the most recent opportunity to wash their hands but washed on the previous opportunity, … and (1 – *c*)^i^(*ρ* + *iγ*) for those who have not washed their hands at all on that day. The total number of HAIs transmitted on the *i*^*th*^ visit of day n is given by

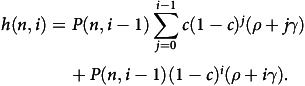




The number of patients without an HA-BSI is reduced by *h(n,i)* and the number of patients with an HA-BSI is increased by *h*(*n,i*) after visit *i.* Additionally, it was assumed that there is a discharge rate of patients without an HA-BSI, represented by δ_*H*_, and a discharge rate for individuals with an HA-BSI represented by δ_*I*_ with δ_*I*_ < δ_*H*_. We assume 1/δ_*H*_ = 10.4 days while 1/δ_*I*_ = 20.1 days. This is an average of estimates from multiple studies.^1,10,11,12,13^ We assume that hospital beds are full through the year, so when a patient is discharged on a particular day a new patient is admitted to the hospital as a patient without an HAI. Thus the number of patients without an HA-BSI at the start of day n *+ 1* is *P*(*n*, *k*) + *H*(*n*, *k*)*δ_I_*. The total annual number of HA-BSIs is found by 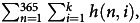 and the total number of patients each year is given by 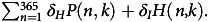


We estimated the net costs of implementing a badge system by examining different improved compliance rates. The costs are based on implementing the system within a hospital, as well as the costs of dealing with particular HA-BSIs within the healthcare system. The net costs are calculated to be the difference between implementation and upkeep of the badge added to the base number of HA-BSIs with the cost savings of preventing a number of HA-BSIs with a higher compliance rate.

The base probability of getting an HA-BSI, *ρ*, and the additive effect of missing hand washing opportunities, *γ*, were estimated from Knudsen and Moller.^14,15^ Their study found that with a compliance rate of 39% and 30% for caregivers and doctors, respectively, there was an incidence rate of 14 HA-BSIs per 10,000 patient days. Once a badge system with feedback was implemented, the compliance rate improved to 71% and 52% for caregivers and doctors, respectively, and resulted in 5 HA-BSIs per 10,000 patient days. The model combined doctors and caregivers compliance rates to estimate a single compliance rate. We used a 9:1 weighted average of the caregiver’s compliance to the doctor’s compliance. This resulted in a preintervention compliance rate of 38.1%, and a postintervention compliance rate of 69.1%.

To estimate *ρ a*nd γ from the 14 HA-BSI to 5 HA-BSI per 10,000 patient days we employed a parameter-search

routine with the model and obtained a *ρ* of 5.9 x 10^–6^ and γ of 4.43 x 10^–5^. These are based on assuming there are 20 handwashing opportunities, estimated from From-Hansen et al., per day per patient for transmitting a BSI,^16^ which was done by taking the number of observations and dividing by the number of beds and days over which the study occurred. Finally, we estimate the cost of each HA-BSI. Yu *et al*. estimate the costs for CLABSI and non-CLABSI HAIs to range from $25,207–55,001.^17^ Reed and Kemmerly estimated the cost of BSI to be $36,441.^18^ Zimlichman *et al*. estimate the cost to be $45,814, and Oliveira *et al*. estimated the costs to be $8,380.^19,20^ We use a recent estimate by Bezerra *et al*. for ICU acquired BSIs of $14,245,^21^ which is likely an underestimate of the real savings.

The costs of implementing the badge system were estimated from Bailey *et al*.^22^ The annual costs were estimated based on the number of beds with the low range of cost ($40,000) used for the least number of beds (100), and the highest costs ($60,000) used for the largest number of beds (1,000). A linear interpolation was used to estimate the costs for the other number of beds considered. Additionally, we use Bailey *et al*. to estimate the startup costs of implementing the system, which are only applied in the first year.^22^ We choose the high value of $2,600 to achieve a conservative estimate in savings.

## Results

To estimate the yearly number of HA-BSIs, the model was iterated for 180 days in order to achieve steady state, then the number of HA-BSIs were estimated over the next 365 days. These calculations were done over a range of the number of beds that the badge system would cover, which describe different size hospitals or possibly the implementation over different areas of a hospital.

**Figure 1.**
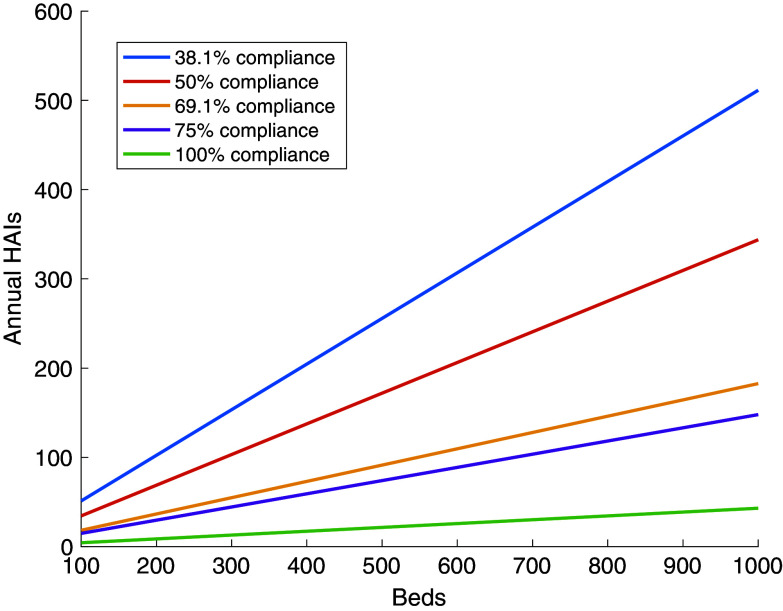
Number of HAIs under different compliance rates over different numbers of beds.

Figure 1 shows the number of HA-BSIs that occur over a year varying different compliance rates and the number of beds. Clearly as the number of beds increase so do the number of HA-BSIs, and as the compliance rate increases the number of HA-BSIs decreases. We focus on a minimal compliance rate of 38.1% that was based on the work of Knudsen and Moller, and a highest compliance rate of 100%, though in general that is not achievable.^14^ We see that with 100 beds there are over 51 HA-BSIs at a 38.1% compliance rate but drops to under 5 at a 100% compliance rate. Increasing the number of beds by 10-fold approximately increases the number of HA-BSIs also by 10-fold to 511 and 43 at 38.1% and 100% compliance rates, respectively.

**Figure 2.**
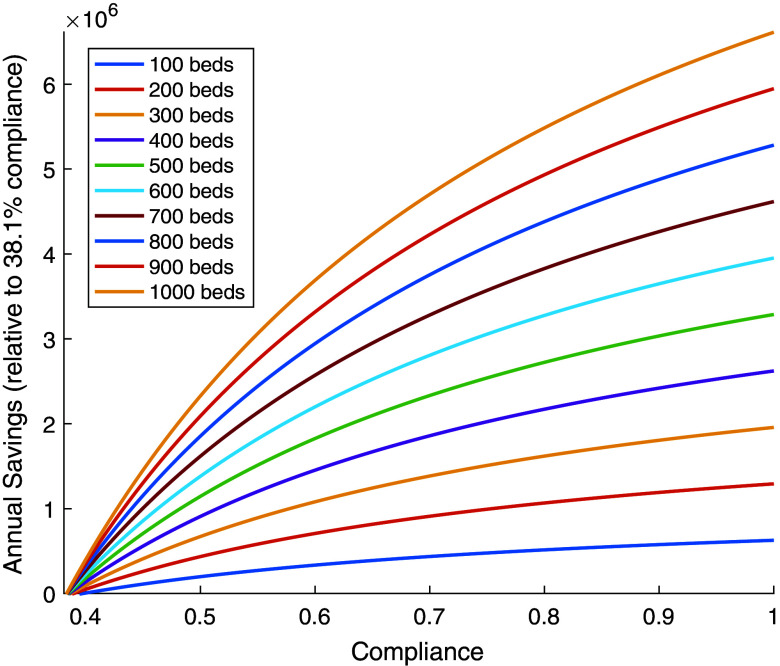
The net savings for implementation based on the compliance rate and number of beds.

On the other hand, Figure 2 shows the amount of health care costs saved based on the number of beds and the compliance rate. The costs are made up of two components where the first cost is based on the upkeep of the system which varies between $40,000 and $60,000 based on the number of beds. The second component is the costs due to the HA-BSIs. Estimates of this cost vary in the literature, though we chose to use an estimate on the lower end of the range. The overall savings for a given number of beds and compliance rate were then computed by subtracting the cost of upkeep the program for the given number of beds to the difference in the costs of HAIs between 38.1% and the given compliance rate.

We observe noticeable savings at each number of beds considered, with savings increasing with increases in compliance rates. The cost of the badge system is quickly recovered, requiring a reduction of only 3–5 HAIs depending on the number of beds. For 100 beds we see a savings of $199,000 for an increase of compliance from 38.1% to 50%, and a savings of $577,000 when that compliance rate improves to 90%. For 1,000 beds we see a savings of $2,300,000 for the increase of 38.1% to 50%, and a saving of over $6,100,000 when the compliance rate increases to 90%. If we also assume a onetime implementation cost of $2,600 per bed, then in the first year the compliance needed to break even is 54.1% for 100 beds and 51.8% for 1,000 beds.

## Discussion

In this study we created a Markov chain model for the transmission of HA-BSIs in the hospital setting where the rate of transmission increases for each consecutive handwashing opportunity that is missed. This model was then parametrized using data from a published study, and then simulated to estimate the yearly saving of implementing a badge system to improve (HH) compliance rates.

Overall, the yearly savings were noticeable even with a smaller number of targeted beds. With 100 targeted beds there was an average savings of about $101,000 per 10% increase in the compliance rate from the baseline of 38.1%, where this increased to about $1,068,000 per 10% increase in the compliance rate for 1,000 targeted beds.

There are some limitations to this study. We based our overall improvement numbers on a single study that observed a significant decrease in HA-BSIs based on the badge system to increase handwashing compliance. However, there are very few sources that give these specific numbers, and a different institution may achieve different results. We also note that part of this study’s data collection occurred during the initial months of Covid. We also assumed that the average number of visits by a healthcare worker as well as handwashing opportunities daily were about 20 for each patient.^16^ This again was the best found in the literature though other institutions’ numbers could vary from this. Finally, the average costs of a HA-BSI vary throughout the literature; though we chose to use an estimate on the lower end of cited numbers, an institution where costs of HAIs are higher could achieve different results with potentially higher savings.
